# Efficient Production of Human Norovirus-Specific IgY in Egg Yolks by Vaccination of Hens with a Recombinant Vesicular Stomatitis Virus Expressing VP1 Protein

**DOI:** 10.3390/v11050444

**Published:** 2019-05-16

**Authors:** Yang Zhu, Yuanmei Ma, Mijia Lu, Yu Zhang, Anzhong Li, Xueya Liang, Jianrong Li

**Affiliations:** 1 Department of Veterinary Biosciences, College of Veterinary Medicine, The Ohio State University, Columbus, OH 43210, USA; yang.zhu@uq.net.au (Y.Z.); ma.194@osu.edu (Y.M.); lu.1383@osu.edu (M.L.); zhang.707@buckeyemail.osu.edu (Y.Z.); li.7875@buckeyemail.osu.edu (A.L.); liang.135@osu.edu (X.L.); 2 Program in Food Science and Technology, The Ohio State University, Columbus, OH 43210, USA

**Keywords:** Human norovirus, IgY, vesicular stomatitis virus (VSV)-based vaccine

## Abstract

Human norovirus (HuNoV) is responsible for more than 95% of outbreaks of acute nonbacterial gastroenteritis worldwide. Despite major efforts, there are no vaccines or effective therapeutic interventions against this virus. Chicken immunoglobulin Y (IgY)-based passive immunization has been shown to be an effective strategy to prevent and treat many enteric viral diseases. Here, we developed a highly efficient bioreactor to generate high titers of HuNoV-specific IgY in chicken yolks using a recombinant vesicular stomatitis virus expressing HuNoV capsid protein (rVSV-VP1) as an antigen. We first demonstrated that HuNoV VP1 protein was highly expressed in chicken cells infected by rVSV-VP1. Subsequently, we found that White Leghorn hens immunized intramuscularly with rVSV-VP1 triggered a high level of HuNoV-specific yolk IgY antibodies. The purified yolk IgY was efficiently recognized by HuNoV virus-like particles (VLPs). Importantly, HuNoV-specific IgY efficiently blocked the binding of HuNoV VLPs to all three types (A, B, and O) of histo-blood group antigens (HBGAs), the attachment factors for HuNoV. In addition, the receptor blocking activity of IgY remained stable at temperature below 70 °C and at pH ranging from 4 to 9. Thus, immunization of hens with VSV-VP1 could be a cost-effective and practical strategy for large-scale production of anti-HuNoV IgY antibodies for potential use as prophylactic and therapeutic treatment against HuNoV infection.

## 1. Introduction

The virus family *Caliciviridae* contains five established genera (*Norovirus*, *Sapovirus*, *Lagovirus*, *Vesivirus*, and *Nebovirus*) and at least six proposed genera (*Recovirus*, *Valovirus*, *Bavovirus*, *Nacovirus*, *Minovirus*, and *Salovirus*) that infect many different animal species including humans. Most of these agents are enteric pathogens whose replication and chief clinical manifestations are gastroenteritis and potentially life-threatening diarrhea. Examples of these viruses include human norovirus (HuNoV), porcine norovirus, bovine norovirus, human sapovirus, porcine sapovirus, and the recently discovered Tulane virus. HuNoV is the major food- and water-borne virus that accounts for more than 95% of nonbacterial acute gastroenteritis worldwide, but this percentage may be underestimated due to the large number of asymptomatic HuNoV infections and lack of proper detection methods [1,2,3,4]. In addition, HuNoV is responsible for over 50% of the outbreaks of foodborne illnesses in the USA [5]. The symptoms often involve projectile vomiting, diarrhea, nausea, and low-grade fever [1,2,6]. HuNoV is transmitted primarily through the fecal–oral route, either by direct person-to-person contact or by fecally contaminated food or water. Although HuNoV infection is usually self-limited disease, it is highly contagious, and only a few particles are thought to be sufficient to cause an infection [1,2,7]. Currently, the National Institute of Allergy and Infectious Diseases (NIAID) classify HuNoV and other caliciviruses as category B priority biodefense pathogens.

Unfortunately, researches on HuNoVs have been hampered due to the fact that it cannot efficiently be grown in cell culture system and lack a robust small animal model for infectivity and pathogenesis study [1,2,4]. Recently, two separate groups in the US reported the replication of HuNoV in cell culture. Jones et al. showed that HuNoV can be grown in human B cells, and that commensal bacteria (such as *Enterobacter cloacae*) facilitate such replication [8]. Ettayebi et al. reported that multiple HuNoV strains can replicate in stem cell-derived human enteroids [9]. Although these studies are highly promising, the robustness of these culture systems needs to be further validated. Despite major efforts, there are still no FDA-approved vaccines or antiviral drugs are available for HuNoV. Recent epidemiological studies found that severe clinical outcomes including death are often associated with high-risk populations such as the elderly, children, and immunocompromised individuals [5,10,11,12]. From 1999 to 2007 HuNoV caused, on average, 797 deaths per year in the USA. Mortality of HuNoV associated infection increases during the epidemic seasons and the burden of HuNoV is much greater in the developing world. The CDC estimates that HuNoV causes the death of 200,000 children under the age of five every year in developing countries. Therefore, there is an urgent need to develop an efficacious vaccine and therapeutic agent for control and prevent HuNoV. 

Antibody-based passive immunization has been shown to be an effective strategy to prevent and treat infectious diseases [13]. Also, rapid and immediate protection can be achieved after passive immunization, for example, against agents of bioterrorism [14]. Using mammalian serum to produce antibodies for oral administration has been described previously [15]. However, its application is limited by the high cost of large-scale antibody production and time-consuming [16,17]. In addition, passive immunization with polyclonal antibodies has also been shown to have higher levels of protection compared to monoclonal antibodies [18]. Immunoglobulin Y (IgY)—the egg yolk antibodies generated as a passive immunity to embryos and baby chicks [19]—can be a good alternative for large-scale production of polyclonal antibodies at a lower cost. Chicken IgYs are transferred from blood to the egg yolk during embryo development [16]. IgY can be easily produced and purified with high yields from egg yolks of immunized hens. In addition, production of antibodies via laying hens only requires the collection of eggs, and the animal number can be reduced due to the high and long-lasting titers produced in chickens [18,19]. Therefore, the IgY technology is a safe, convenient, and inexpensive strategy to prevent and control infectious diseases, especially for gastrointestinal infections [20,21].

Previous reports have shown that HuNoV-specific IgY could be produced by immunization of hens with HuNoV virus-like particles (VLPs) or P particles, which were purified from insect cells using a baculovirus expression system [18,22]. In addition, these IgYs can block the binding of HuNoV VLPs to its receptors, the histo-blood group antigens (HBGA), suggesting that IgY may potentially be used as passive immunization and therapeutic agent for HuNoV. Although these results are promising, purification of VLPs from insect cells is time-consuming and expensive which may limit the large-scale production of IgY. Previously, our laboratory developed a more efficient, convenient, and economical strategy to produce HuNoV VLPs [23,24]. We constructed a recombinant vesicular stomatitis virus expressing VP1 gene of a HuNoV GII.4 strain (rVSV-VP1). The VLP production by rVSV-VP1 was significantly higher than that by insect cells-baculovirus expression system. Vaccination of mice with rVSV-VP1 triggered significantly higher HuNoV-specific humoral, cellular, and mucosal immunities than traditional VLP vaccination [23,24].

In this study, we developed a highly efficient bioreactor for large-scale production of chicken egg yolk IgY antibodies using rVSV-VP1 as an antigen. We found that HuNoV VP1 was highly expressed in chicken cells infected by rVSV-VP1 and that hens vaccinated rVSV-VP1 induced a high level of HuNoV-specific IgY in egg yolks. Interestingly, intramuscular vaccination of rVSV-VP1 produced approximately three times more HuNoV-specific IgY than combination of intramuscular and nasal drop vaccination route. Importantly, HuNoV-specific IgY produced by rVSV-VP1 vaccination was capable of blocking the binding of HuNoV VLPs to type A, B, and O HBGA receptors. In addition, egg yolk IgY antibodies remained stable at temperature below 70 °C and at pH ranging from 4 to 9. These data demonstrated that recombinant rVSV-VP1 is a highly effective antigen for large-scale production of HuNoV-specific IgY for passive immunization and therapeutic agent.

## 2. Materials and Methods

### 2.1. Virus and Cell Culture

Recombinant vesicular stomatitis virus (rVSV) expressing human NoV capsid protein (rVSV-VP1) was previously constructed in our laboratory [23]. Working stocks of rVSV-VP1 were propagated in confluent BSRT7 cells. Briefly, BSRT7 cells in T150 flask were infected by rVSV-VP1 at a multiplicity of infection (MOI) of 10. After 1 h of absorption, the inoculum was removed, and the cells were washed twice with Dulbecco’s modified Eagle’s medium (DMEM). After addition of 15 mL fresh DMEM (supplemented with 2% fetal bovine serum), the infected cells were incubated at 37 °C in CO_2_ incubator. When extensive cytopathic effects (CPE) were observed, cell culture fluid was harvested, and virus titer was determined by plaque assay in Vero cells. 

### 2.2. Multistep Growth Curves in BSRT7 and Chicken DF-1 Cells

Thirty-five-millimeter dishes were seeded with BSRT-7 or DF-1cells (kindly provided by Dr. Qingzhong Yu at USDA ARS, Athens, GA) and were infected with rVSV-VP1 at a multiplicity of infection (MOI) of 10. After 1 h of absorption, the inoculum was removed, the cells were washed twice with DMEM, 2 mL of fresh DMEM (supplemented with 2% fetal bovine serum) was added, and the infected cells were incubated at 37 °C. At the indicated intervals, 500-µL aliquots of the cell culture fluid were removed and the same amount of fresh DMEM was added back to the virus-infected cells. Virus titers were determined by plaque assay in Vero cells. 

### 2.3. Determination of the Kinetics of VP1 Expression in Cell Culture

Six-well plates were seeded by BSRT7 or DF-1cells (2 × 10^5^ cells per well). After 24 h incubation at 37 °C in CO_2_ incubator the cells were infected with rVSV-VP1 at an MOI of 10. At the indicated time points, cells were lysed in lysis buffer containing 5% β-mercaptoethanol, 0.01% NP-40, and 2% sodium dodecyl sulfate (SDS). Proteins were separated by 12% SDS-PAGE and transferred to a Hybond ECL nitrocellulose membrane (Amersham, Piscataway, NJ, USA) in a Mini Trans-Blot electrophoretic transfer cell (Bio-Rad, Hercules, CA, USA). The blot was probed with guinea pig anti-human NoV VP1 antiserum (a generous gift from Dr. Xi Jiang) at a dilution of 1:6000, followed by horseradish peroxidase-conjugated goat anti-guinea pig IgG secondary antibody (Santa Cruz Biotechnology, Inc., Dallas, TX, USA) at a dilution of 1:20,000. The blot was developed with SuperSignal West Pico Chemiluminescent Substrate (Thermo Scientific, Waltham, MA, USA) and exposed to Kodak BioMax MR film (Kodak, Rochester, NY, USA). The harvested cell lysates were also subjected to Western blot using anti β-actin antibody (Proteintech, Rosemont, IL, USA), which recognizes β-actin from multiple species, including human, mouse, hamster, and chicken. The protein bands were scanned and quantified using a Typhoon PhosphorImager and ImageQuant TL software (GE Healthcare, Piscataway, NJ, USA). The VP1 protein band of each time point was normalized by the respective β-actin band. The time point (12 h postinfection in BSRT7 cells) with highest VP1 expression was set as 100%. The percentage of VP1 expression at other time points was normalized by the VP1 expression at 12 h postinfection in BSRT7 cells. 

### 2.4. Chickens and Immunization

The animal study was conducted in strict accordance with USDA regulations and the recommendations in the Guide for the Care and Use of Laboratory Animals of the National Institutes of Health, and was approved by The Ohio State University Institutional Animal Care and Use Committee (animal protocol no. 2013A00000011). Chickens were housed in cages inside high-security isolation rooms provided with HEPA-filtered intake and exhaust air at The Ohio Agriculture Research and Development Center, The Ohio State University. The animal care facilities at The Ohio State University are AAALAC accredited. Before animal study, blood samples were collected from each chicken to confirm that they were negative for HuNoV antibody.

Six, 21-week-old, healthy White Leghorn chickens were provided by Dr. Lilburn, Department of Animal Sciences, The Ohio State University and were randomly divided into two groups (three chickens per group). Prior to the study, these chickens were negative for VSV and HuNoV antibody. Chickens in Group I were immunized intramuscularly by injecting 500 µL of DMEM containing 5 × 10^7^ PFU of rVSV-VP1 into three different locations of the pectoral muscle. Chickens in Group II were immunized by combination of intramuscular and intranasal routes. Specifically, 300 µL of rVSV-VP1 (3 × 10^7^ PFU) was injected into three different locations of the pectoral muscle, and the remaining 200 µL of rVSV-VP1 (2 × 10^7^ PFU) was used for nasal drop vaccination. At week 2 postimmunization, chickens in groups I and II were boosted with 5 × 10^7^ PFU of rVSV-VP1 via intramuscular and combination of intramuscular and intranasal routes, respectively. After immunization, eggs were collected daily until week 4 post-booster vaccination. In addition, hens were observed daily for any abnormal reactions. Eggs that were collected one week before immunization were used as negative control. Eggs were stored at 4 °C before IgY extraction.

### 2.5. Production and Purification of HuNoV VLPs by a Baculovirus Expression System

Purification of VLPs from insect cells was described previously with some minor modifications [23]. *Spodoptera frugiperda* (Sf9) cells were infected with baculovirus expressing HuNoV VP1 at an MOI of 10, and the infected Sf9 cells and cell culture supernatants were harvested at 6 days postinoculation. The VLPs were purified from cell culture supernatants and cell lysates by ultracentrifugation through a 40% (*w*/*v*) sucrose cushion, followed by CsCl isopycnic gradient (0.39 g/cm^3^) ultracentrifugation. Purified VLPs were analyzed by sodium dodecyl sulfate–polyacrylamide gel electrophoresis (SDS-PAGE) followed by Coomassie blue staining. The protein concentrations of the VLPs were measured by using the Bradford reagent (Sigma Chemical Co., St. Louis, MO, USA).

### 2.6. Extraction and Purification of IgY from Chicken Egg Yolks

IgY was extracted and purified from egg yolks using polyethylene glycol 8000 (PEG 8000, Sigma, St. Louis, MO, USA) precipitation method [25] with some modifications. Briefly, egg yolks were diluted in three volumes of PBS (pH 7.4) and mixed, and PEG 8000 was added to a final concentration of 3.5%. After vortexing, the mixture continued to roll on a rolling mixer for 20 min. The mixtures were centrifuged at 13,000× *g* for 20 min at 4 °C, and the precipitated debris were removed. Subsequently, PEG 8000 was added to the supernatant to a final concentration of 8.5%, and the samples were mixed on a rolling mixer for 20 min. The mixtures were centrifuged again at 13,000× *g* for 20 min at 4 °C. The precipitated pellets containing IgY were dissolved in 10 mL of PBS and then precipitated again with 12% of PEG 8000 using the same procedures described above. The final pellets was dissolved in 2.0 mL of PBS, filtered through a 0.45 µm filter, and stored at −20 °C. The purity of the IgY was determined by SDS-PAGE followed by Coomassie blue staining. 

### 2.7. Determination of HuNoV-Specific IgY and Total IgY Titers in Egg Yolk

Standard ELISA measured HuNoV-specific IgY antibody titers. Briefly, 96-well microtiter plates were coated with 100 μL of purified HuNoV VLP antigen (200 ng/well) and incubated overnight at 4 °C. After blocking with 5% nonfat milk, 10 times serially diluted chicken IgYs were added to the antigen-coated wells and incubated at 37 °C for 1 h. After washing with PBST (PBS containing 0.05% Tween), goat anti-chicken IgY-HRP (1:5000) (Santa Cruz Biotechnology) was added for 1 h. Plates were washed and developed with 75 µL of 3,3’,5,5’-tetramethylbenzidine (TMB), and the optical density (OD) at 450 nm was determined using an enzyme-linked immunosorbent assay (ELISA) plate reader. The IgYs from pre-immunized chicken yolks were used as controls. To calculate the amount of total IgY and HuNoV-specific IgY, a standard curve was set-up: wells were coated with 100 μL of serially diluted pure chicken IgY (Promega, Madison, WI, USA) at a concentration from 0.0075 μg/mL to 1 μg/mL. After washing with PBST, 100 μL of goat anti-chicken IgY-HRP (Santa Cruz Biotechnology) at a dilution of 1:1000) were added and incubated at 37 °C for 1 h. The bound HRP was colorized by substrate reagent (Kirkegaard and Perry Laboratories, Inc., Gaithersburg, Maryland, USA), followed by a reading of the signal intensity at 450 nm (Epoch Micro-Volume Spectrophotometer System, BioTek, Winooski, VT, USA). The resulting standard curve of absorbance was used to quantify the relative concentration of total IgY and HuNoV-specific IgY from the egg yolks by coating plates with either HuNoV VLP or rabbit anti-chicken IgY antibodies (10 μg/mL, Sigma) to capture the total IgY or HuNoV-specific IgY.

### 2.8. Analysis of Chicken IgY by SDS-PAGE

The purified IgYs from egg yolks were analyzed by SDS-PAGE. Samples were boiled for 5 min in loading buffer containing 1% SDS, 2.5% β-mercaptoethanol, 6.25 mM Tris-HCl (pH 6.8), and 5% glycerol and loaded onto a 12% polyacrylamide gel. Proteins were visualized by Coomassie blue staining.

### 2.9. Characterization of IgY by Western Blot Analysis

Specific reactions of the chicken IgY with HuNoV capsid protein were examined by Western blot analysis. The purified HuNoV virus-like particles (VLPs) were separated by conventional 12% SDS-PAGE and transferred to a Hybond ECL nitrocellulose membrane (Amersham) in a Mini Trans-Blot electrophoretic transfer cell (Bio-Rad). After blocking with 5% nonfat milk, the membrane was incubated with anti-HuNoV-specific IgY or nonspecific IgY (1:1000) in 1% nonfat milk-PBS at 4 °C overnight, followed by horseradish peroxidase (HRP)-conjugated goat anti- chicken IgY secondary antibody (Santa Cruz Biotechnology) at a dilution of 1:5000. The blot was developed with SuperSignal West Pico chemiluminescent substrate (Thermo Scientific) and exposed to Kodak BioMax MR film (Kodak).

### 2.10. HBGA Binding Assay

The saliva-based HBGA binding and blocking assays were performed as described previously [18,26,27]. To avoid potential HuNoV-specific antibodies in the saliva that may interfere with the receptor-binding assay, saliva samples were boiled before being used in the assays. The boiled human saliva samples with known HBGA phenotypes (A, B, or O) were diluted 1000-fold and coated on 96-well microtiter plates at 4 °C overnight. After blocking with 5% nonfat milk in PBS, HuNoV VLPs were added to a final concentration of 4 μg/mL. The bound VLPs were detected by using serially diluted IgYs (from 1:1000 to 1:128,000), followed by the addition of HRP-conjugated goat anti-chicken IgY at a dilution of 1:5000. The color was then developed by adding tetramethylbenzidine peroxidase liquid substrate (Kirkegaard and Perry Laboratory) and stopped after 10 min of incubation at 22 °C by adding 1 mol/L sulfuric acid. Optical density (OD) was measured at 450 nm with the use of an Epoch Micro-Volume Spectrophotometer System (BioTek, Winooski, VT, USA).

### 2.11. HBGA Blocking Assay

HBGA blocking assay was performed to determine the inhibitory activity of IgY against the binding of HuNoV VLPs to the HBGA antigens [28]. The boiled human saliva samples with known HBGA phenotypes (A, B, or O) were diluted 1000-fold and coated on 96-well microtiter plates at 4 °C overnight. The HuNoV VLPs were preincubated with serially diluted IgYs for 1 h at 37 °C, and IgY-VLP solutions were added to the saliva-coated wells. Plates were washed 3 times with 0.1 mol/L sodium phosphate buffer (pH, 6.4). Then, a guinea pig anti-HuNoV VLP antiserum at a dilution of 1:1000 was added and incubated for 1 h at 4 °C. The plates were washed again, and HRP-conjugated goat anti-guinea pig IgG (at dilution of 1:5000) was added and incubated for 1 h at 4 °C. The color was then developed by adding tetramethylbenzidine peroxidase liquid substrate (Kirkegaard and Perry Laboratory) and stopped after 10 min of incubation at 22 °C by adding 1 mol/L sulfuric acid. The blocking rates were calculated by comparing the optical densities (ODs) measured with and without blocking by the chicken IgYs. The IgYs from chickens before immunization were used as controls [26]. Blank wells were incubated with buffer instead of IgY-VLP and served as negative control whereas VLP binding to carbohydrates in the absence of IgY sample was used as a positive control.

### 2.12. The Effects of pH on the Stability of HuNoV-Specific IgY

For the pH stability, the purified total IgY solution (1 mL, 1:100, pH 7.4) was diluted in 0.1 mol/L sodium phosphate buffer (pH 6.4). The pH of the solution was adjusted using either HCl or NaOH to a final pH ranging from 2 to 11. The solution was incubated at 37 °C for 3 h, followed by neutralization by adding 5 × PBS (pH 6.4) to a final pH of 7. The HBGA blocking assays were performed to measure the activity of IgY, as described above.

### 2.13. The Effects of Heat Treatment on the Stability of HuNoV-Specific IgY

To determine heat stability of HuNoV-specific IgY, the purified total IgY solution (1 mL, 1:100, pH 7.4) was treated at temperature ranging from 4 to 80 °C for up to 30 min. After heat treatment, the samples were cooled quickly on ice. The HBGA blocking assays were performed to measure the activity of IgY as described above.

### 2.14. Statistical Analysis

Quantitative analysis was performed by either densitometric scanning of autoradiographs or by using a Typhoon PhosphorImager and ImageQuant TL software (GE Healthcare, Piscataway, NJ, USA). Each experiment was performed three to six times. Statistical analysis was performed by one-way multiple comparisons using SPSS software 13.0 (SPSS, Chicago, IL, USA). A *p*-value of <0.05 was considered statistically significant.

## 3. Results

### 3.1. HuNoV VP1 Is Highly Expressed in Chicken Cells

**Figure 1:**
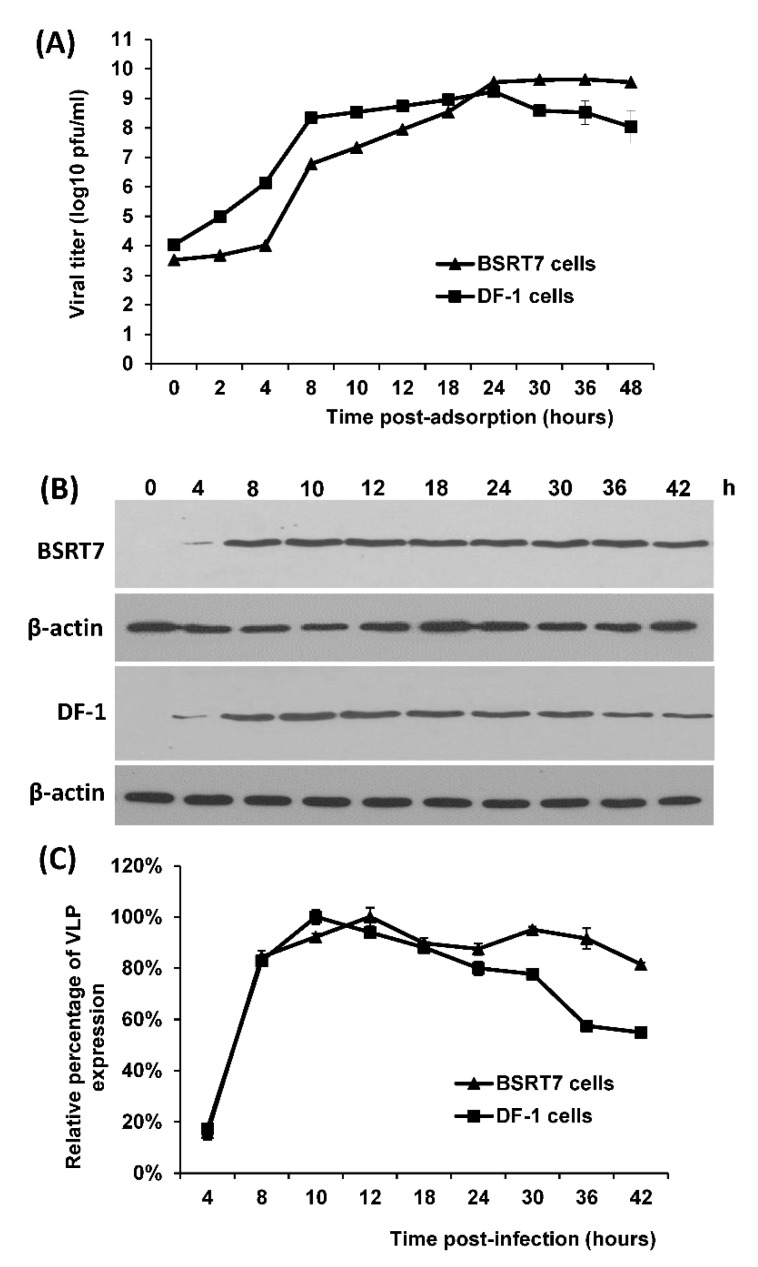
The growth kinetics and virus-like particle (VLP) expression of rVSV-VP1 in BSRT7 and DF-1 cells. (**A**) Growth kinetics of rVSV-VP1. Confluent BSRT7 and DF-1 cells were infected with rVSV-VP1 at a multiplicity of infection (MOI) of 10. After 1-h incubation, the inoculum was removed, the cells were washed with DMEM, and fresh medium (containing 2% fetal bovine serum) was added, followed by incubation at 37 °C. Samples of supernatant were harvested at the indicated intervals over a 48-h time period, and the virus titer was determined by plaque assay. (**B**) Dynamics of VP1 expression in cell lysates by Western blot. BSRT-7 and DF-1 cells were infected with rVSV-VP1 at an MOI of 10. Cytoplasmic extracts were harvested at indicated time points. Equal amounts of total cytoplasmic lysate were analyzed by SDS-PAGE, followed by Western blot analysis using guinea pig anti-HuNoV VP1 antiserum or β-actin antibody. (**C**) Quantitative analysis of VP1 expression. The VP1 and β-actin protein bands were scanned and quantified using a Typhoon PhosphorImager and ImageQuant TL software. The VP1 protein band of each time point was normalized by the respective β-actin band. The time point (12 h postinfection in BSRT7 cells) with highest VP1 expression was set as 100%. The percentage of VP1 expression at other time points was normalized by the VP1 expression at 12 h postinfection. Three independent experiments were used to generate the quantitative analysis shown. Data was expressed as the mean ± the standard deviation.

VSV replicates efficiently in a wide range of mammalian cell lines. We previously showed that rVSV-VP1 replicated to a high titer in BSRT7 cells, baby hamster kidney cells [23]. Before chicken vaccination, we first determined whether rVSV-VP1 replicated efficiently in DF-1 cells, which are derived from chicken embryo. Briefly, BSRT7 and DF-1 cells were infected with rVSV-VP1 at an MOI of 10 and the kinetics of viral replication was determined at time points from 0 to 48 h postinfection. As shown in Figure 1A, rVSV-VP1 replicated efficiently in both DF-1 and BSRT7 cells. Viral titer gradually increased at 2 h postinfection and reached a peak titer of 7.0 × 10^9^ pfu/mL at 24 h postinfection. However, in BSRT7 cells, the virus titer started to increase at 4 h postinfection, and reached a peak titer of 9.6 × 10^9^ at approximately 30 h postinfection. In addition, virus titer in DF-1 cell decreased after 24 h postinfection because of the cell death. Overall, there is no significant difference in viral replication in DF-1 and BSRT7 cells (*p* > 0.05). 

We next examined the kinetics of HuNoV VP1 expression in BSRT7 and DF-1 cells. Briefly, BSRT7 and DF-1 cells were infected with rVSV-VP1 at an MOI of 10 and cell lysates were harvested at the indicated times. The expression of VP1 was determined by Western blot. β-actin was loaded as an internal control. The VP1 expression was detectable at 4 h postinfection in both cell lines, and reached a peak between 10 and 12 h postinfection (Figure 1B). HuNoV VP1 was highly expressed in both BSRT7 and DF-1 cells during 4-30 h postinfection. VP1 expression in DF-1 decreased after 30 h postinfection due to the cell death. Quantitation of three independent experiments showed there were no significant difference in VP1 expression between BSRT7 and DF-1 cells at time points from 4 to 30 h postinfection (*p* > 0.05) (Figure 1C). 

### 3.2. Safety of rVSV-VP1 in Chickens

We previously showed that rVSV-VP1 was not only attenuated in vitro and in vivo, but also triggered a high level of mucosal, humoral, and cellular immunities in a mouse model [23]. Since rVSV-VP1 can grow in chicken cells, we hypothesis that chickens will be susceptible to rVSV-VP1 infection and thus produce HuNoV-specific IgY in eggs. To do this, rVSV-VP1 was inoculated into chickens by two routes: intramuscular or combination of intramuscular and intranasal route. After vaccination, the safety of rVSV-VP1 in chickens was monitored daily. No abnormal reaction was observed in chickens vaccinated by both routes. Recombinant rVSV-VP1 vaccination did not affect feed intake and egg production. This result suggests that rVSV-VP1 was safe to chickens.

### 3.3. Purification and Characterization of HuNoV-Specific IgY from Chicken Yolks

**Figure 2:**
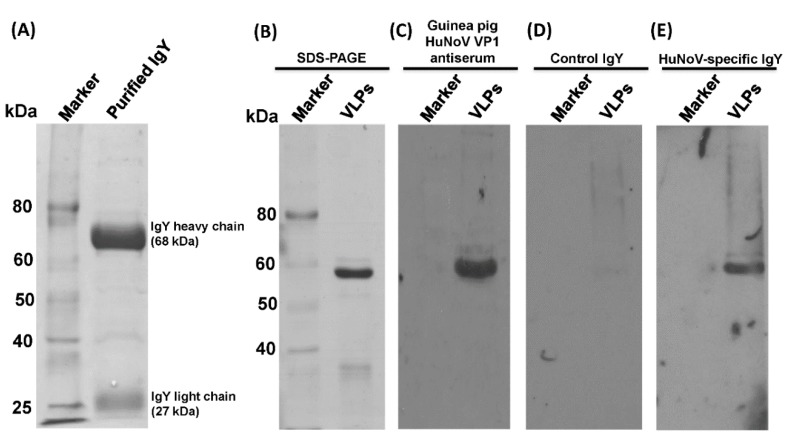
Characterization of total IgY and HuNoV-specific IgY in egg yolks. (**A**) Analysis of total IgY by SDS-PAGE. Total IgY was purified from eggs and analyzed by SDS-PAGE. (**B**) Analysis of purified HuNoV VLPs by SDS-PAGE. VLPs were purified from insect cells infected by baculovirus expressing VP1. Two-hundred nanograms of VLPs was loaded. (**C**) Western blot analysis of HuNoV VLPs using guinea pig anti-HuNoV VP1. 200 ng of VLPs was loaded in SDS-PAGE, and subjected to Western blot using guinea pig anti-HuNoV VP1 as the primary antibody. (**D**) Western blot analysis of HuNoV VLPs using control IgY. Two-hundred nanograms of VLPs was loaded in SDS-PAGE and subjected to Western blot using total IgY purified from eggs collected before rVSV-VP1 vaccination (nonspecific IgY). (**E**) Western blot analysis of HuNoV VLPs using HuNoV-specific IgY. Two-hundred nanograms of VLPs was loaded in SDS-PAGE, and subjected to Western blot using total IgY purified from eggs collected at week 4 postvaccination (HuNoV-specific IgY).

After rVSV-VP1 vaccination, eggs from each hen were collected daily. To determine whether rVSV-VP1 triggers HuNoV-specific IgY, total IgY was purified from each egg collected at weeks 1, 2, 3, and 4 postvaccination. To examine the purity of total IgY, 5 μL of total IgY from one egg collected at week 4 postvaccination was analyzed by SDS-PAGE. As shown in Figure 2A, two protein bands with molecular weight of 68 and 27 kDa were observed, which were consistent with the size of heavy and light chain of IgY, respectively. 

To next determined whether purified total IgYs contain HuNoV-specific IgY. Purified HuNoV VLPs (approximately 56 kDa) were separated by SDS-PAGE (Figure 2B), followed by Western blotting using total IgY or serum IgG. As a positive control, a band of 56kDa protein was detected when anti-HuNoV VP1 serum antibody raised in guinea pig was used (Figure 2C). As a negative control, no protein bands were identified in Western blot using total IgY from eggs prior to rVSV-VP1 vaccination (Figure 2D). As shown in Figure 2E, HuNoV VP1 was detected by Western blot when total IgY from eggs collected at week 4 after rVSV-VP1 vaccination. Thus, these results confirmed that rVSV-VP1 vaccination triggered HuNoV-specific IgY in egg yolks.

### 3.4. Kinetics of HuNoV-Specific IgY Responses in Chicken Egg Yolks after rVSV-VP1 Vaccination.

**Figure 3:**
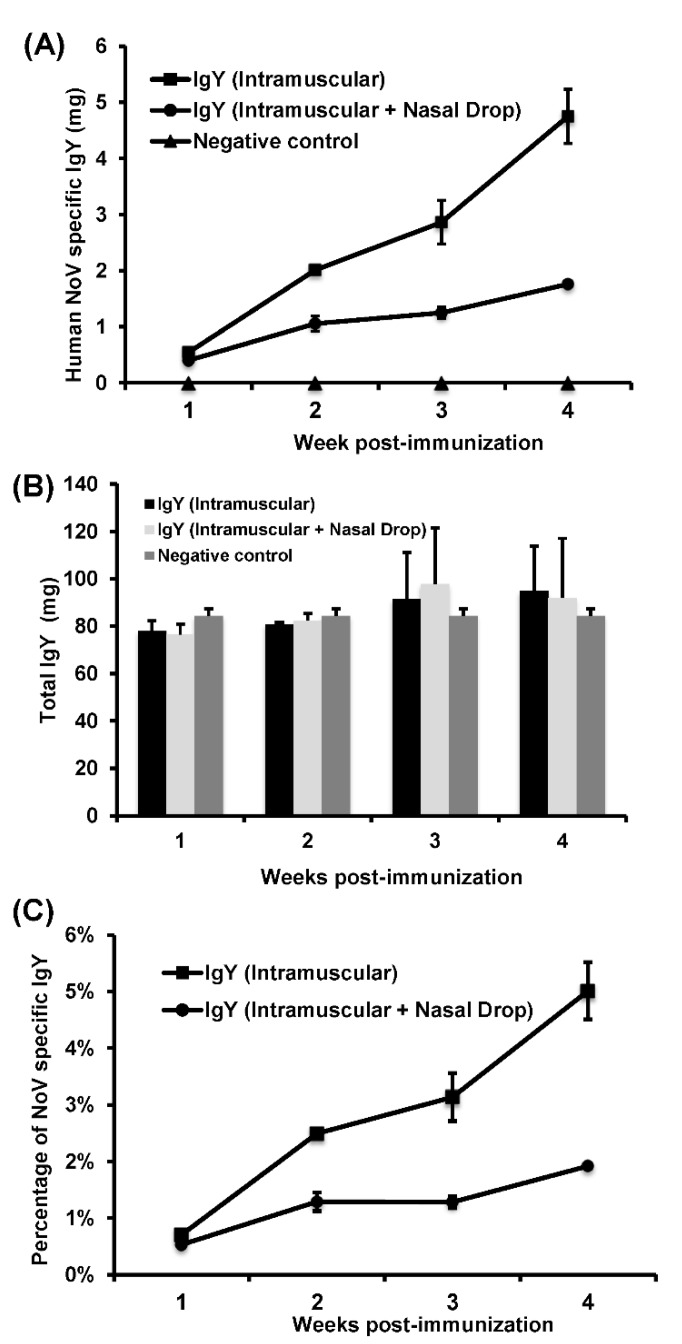
Kinetics of IgY production in chicken vaccinated with recombinant rVSV-VP1. (**A**) The amount of HuNoV-specific IgY in egg yolks. HuNoV-specific IgY in each egg was determined by ELISA as described in Materials and Methods. HuNoV VLPs were used for coating antigens in ELISA. (**B**) The amount of total IgY in egg yolks. Total IgY in each egg was determined by ELISA. A standard IgY product was used to generate standard curve. (**C**) The percentage of HuNoV-specific IgY in total IgY. For each egg, the percentage was calculated based on HuNoV-specific IgY in total IgY.

We next determined the kinetics of HuNoV-specific IgY responses following rVSV-VP1 vaccination. Briefly, 96-well plates were coated with 200 ng of purified HuNoV VLP antigen in each well and were reacted with serially diluted chicken IgY at 37 °C for 1 h. After reacting with HRP labeled goat anti-chicken IgY followed by addition of substrate reagent, an ELISA reader read the OD value at 450 nm. Subsequently, the amount of total IgY and HuNoV-specific IgY was quantified using standard curve generated by commercially available IgY. As shown in Figure 3A, the amount of HuNoV-specific IgY gradually increased after rVSV-VP1 immunization. Interestingly, at weeks 2, 3, and 4 postvaccination, the levels of HuNoV-specific IgY in intramuscular vaccination group were significantly higher than those in the combined intramuscular and nasal drop vaccination group (*p* < 0.05). At week 4 postvaccination, HuNoV-specific IgY in intramuscular group reached 4.8 mg/yolk whereas only 1.8 mg/yolk HuNoV-specific IgY was detected in intramuscular and nasal drop group. 

Using a similar method, the amount of total IgY in each egg yolk was determined. As shown in Figure 3B, there was no significant difference in total IgY between the two vaccination groups (*p* > 0.05). In addition, there was no significant difference in total IgY from eggs collected before and after rVSV-VP1 vaccination (*p* > 0.05). Under our experimental condition, the amount of total IgY in each yolk ranged from 84.4 ± 3.8 to 86.4 ± 7.8 mg. 

Next, we calculated the percentage of HuNoV-specific IgY in total IgY in each egg yolk. As shown in Figure 3C, the percentage of HuNoV-specific IgY gradually increased from 0.7% to 5% in the intramuscular immunization group. In contrast, the percentage of HuNoV-specific IgY ranged from 0.05% to 2% in the combined immunization group. Collectively, these results showed that both immunization routes were capable of producing HuNoV-specific IgY, and intramuscular injection alone was more effective in triggering HuNoV-specific IgY than combination of intramuscular and nasal drop route.

### 3.5. Development of a Saliva-Based HBGA Binding Assay Using Purified IgY as a Detection Antibody

**Figure 4:**
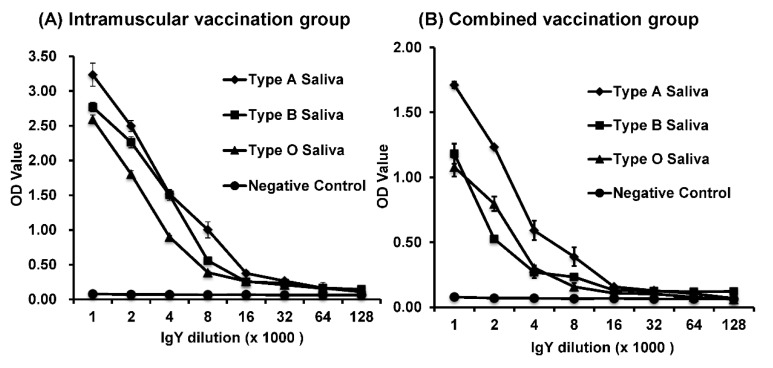
Histo blood group antigen (HBGA) binding assay using purified total IgY as a primary antibody. (**A**) Saliva-based HBGA binding assays using IgYs isolated from intramuscular vaccinated hens. (**B**) Saliva-based HBGA binding assays using IgYs isolated from intramuscular and nasal drop vaccinated hens. Each data point represents an average value of binding assays using IgYs isolated from three eggs ± the standard deviation.

HuNoV utilizes HBGAs as attachment factors. HuNoV VLPs possess authentic receptor binding activity that can be detected by a HBGA binding assay. In this assay, human saliva containing HBGA receptors (types A, B, or O) or synthetic HBGAs can be recognized by HuNoV VLPs, which can be further detected by anti-HuNoV serum and a HRP-conjugated second antibody. Therefore, we determined whether total IgY derived from rVSV-VP1 vaccinated hens could be used as the primary antibody for the HBGA binding assay. Briefly, 96-well plates were coated with type A, B, or O human saliva, and 200 ng of HuNoV VLPs were added to bind the HBGA receptor. After 1-h incubation, a serial dilution of total IgY was added followed by addition of HRP-conjugated goat anti-chicken IgY. After the addition of substrate reagent, an ELISA reader measured OD450. Purified total IgY from rVSV-VP1 vaccinated groups strongly reacted with HuNoV VLPs in the saliva-based HBGA binding assays (Figure 4A,B). In contrast, control IgY from pre-immunized hens was negative in this assay. Overall, total IgY from intramuscular vaccination group had significantly higher OD values than those in combined vaccination group (*p* < 0.05, compare the OD value in Figure 4A,B). In addition, it appears that VLPs had a stronger binding activity to type A saliva compared to types B and O saliva (Figure 4A,B). These results demonstrated that purified total IgY induced by rVSV-VP1 vaccination could be used as a primary antibody to measure binding of HuNoV VLP to all three types of saliva in HBGA binding assays.

### 3.6. IgY Antibodies Blocked the Binding of HuNoV VLPs to the HBGA Receptors

**Figure 5:**
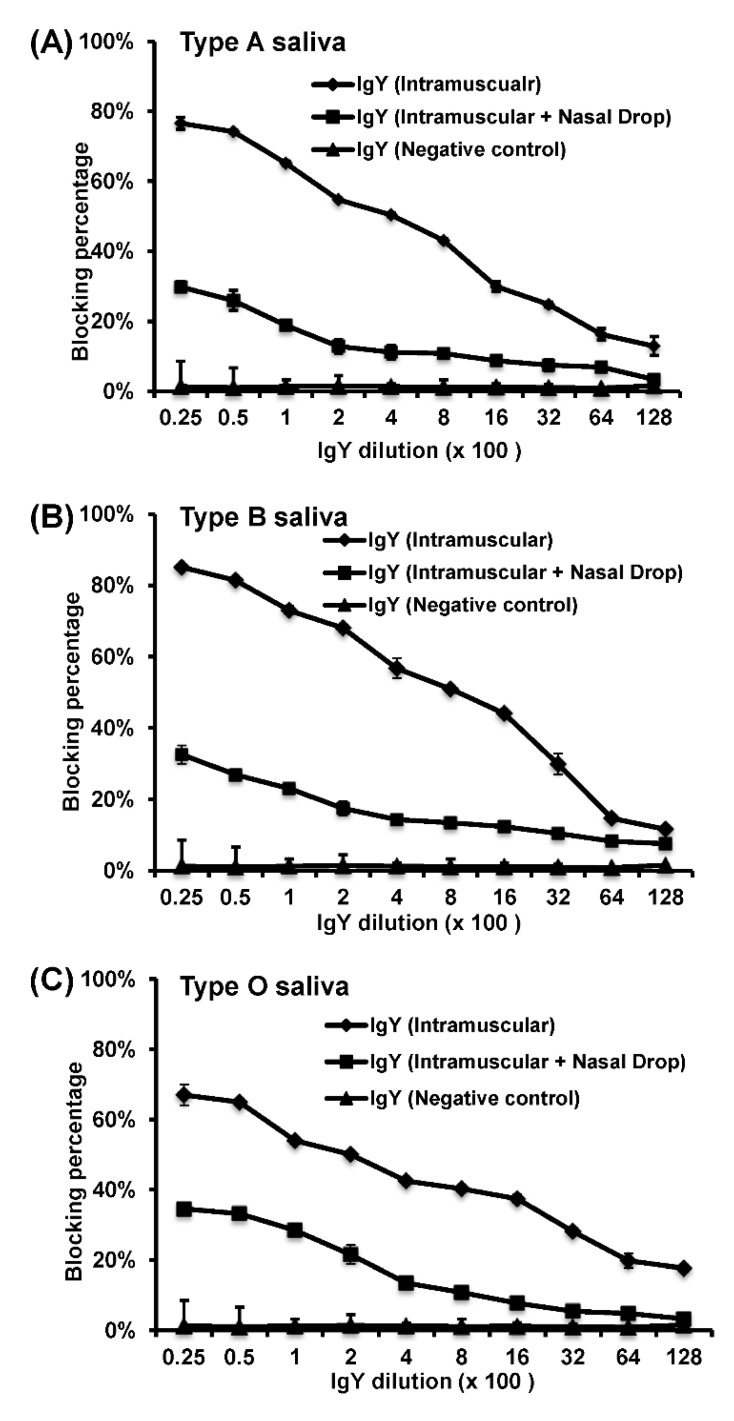
Chicken IgY induced by rVSV-VP1 vaccination blocks the binding of HuNoV VLPs to HBGAs. (**A**) IgY blocked binding of HuNoV VLPs to type A antigen. (**B**) IgY blocked binding of HuNoV VLPs to type B antigen. (**C**) IgY blocked binding of HuNoV VLPs to type O antigen. Data are average of three replicates ± the standard deviation.

Next, we determined whether the purified IgY has potential antiviral activity that can be developed as a therapeutic agent against HuNoV infection. Recently, a HBGA blocking assay, which measures the ability of antibody blocks the binding of HuNoV VLPs to the attachment factors (HBGAs), has been developed [28,29]. Presumably, blockage of viral receptor binding activity will inhibit viral attachment, entry, and subsequent viral infection. Thus, this assay can serve as a useful surrogate assay for serum–virus neutralization assay. Briefly, 96-well plates were coated with a known saliva type (A, B, or O) at 4 °C overnight. The VLPs were preincubated with the serially diluted IgY at 37 °C for 1 h before adding to the saliva-coated wells. Then, a guinea pig anti-HuNoV VLP antiserum was added, followed by the addition of HRP-conjugated goat anti-guinea pig IgG. After the addition of substrate reagent, OD_450_ was measured by an ELISA reader. Percent of blocking activity was calculated by comparing the OD values measured with or without blocking by the chicken IgYs. As shown in Figure 5, total IgY antibodies isolated from egg yolks of rVSV-VP1 vaccinated groups were capable of blocking the binding of HuNoV VLP to HBGAs (A, B, or O antigen) in a dose-dependent manner with a BT50 (a serum dilution with 50% blocking activity) of approximately 1:400, 1:800, and 1:100, respectively. As controls, IgY purified from egg yolks of chickens before immunization did not have detectable blocking activity. Again, total IgY from intramuscular group had a significantly higher blocking activity compared to the intramuscular and nasal drop group (*p* < 0.05). Therefore, IgY from rVSV-VP1 vaccinated hens specifically blocked the binding of HuNoV VLPs to the HBGAs.

### 3.7. Thermal Stability of HuNoV-Specific IgY

**Figure 6:**
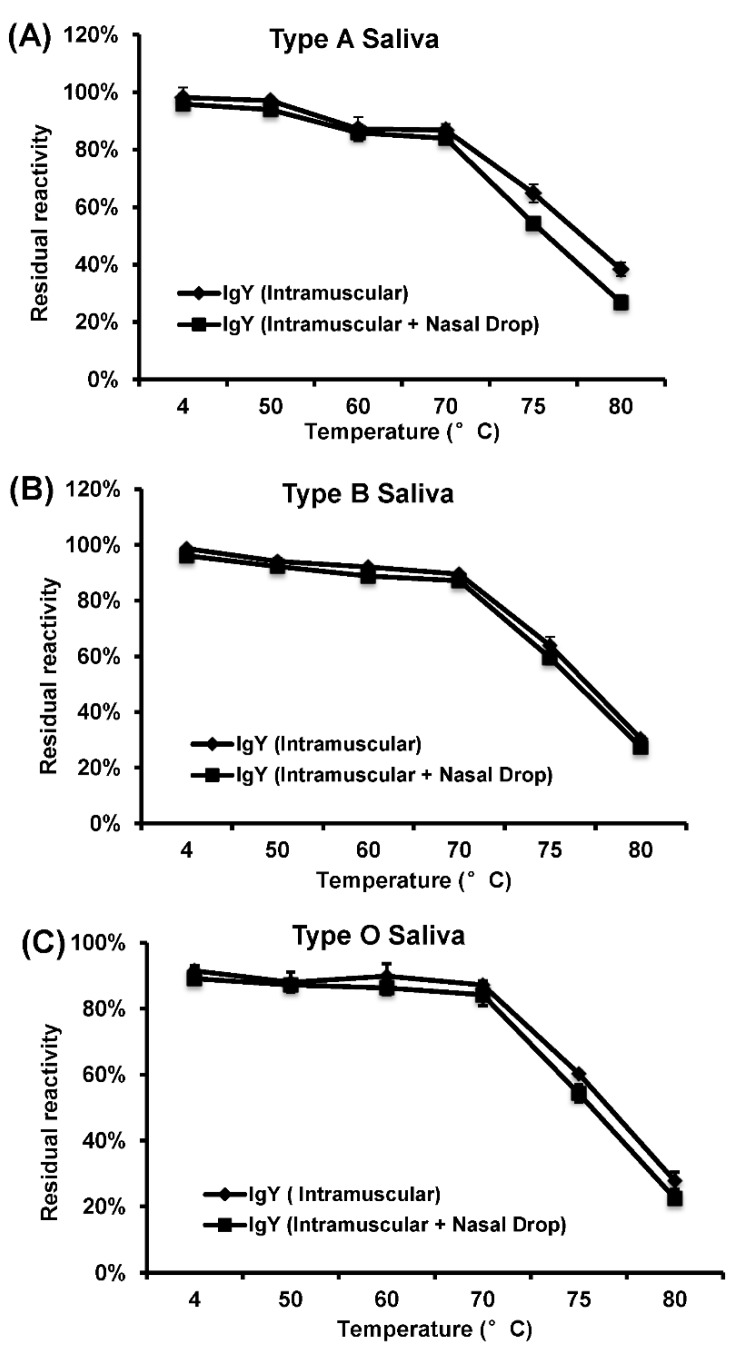
Effects of temperature on the ability of IgY to block the binding of HuNoV VLPs to HBGAs. (**A**) Blockage activity to type A saliva. (**B**) Blockage activity to type B saliva. (**C**) Blockage activity to type O saliva. A percentage (%) of residual blocking activity in comparison with an untreated sample is shown. Each data point represents the average of three replicates ± the standard deviation.

To investigate thermal stability of IgY, IgY solution was incubated at temperature ranging from 4 to 80 °C for up to 30 min, and the receptor blocking activity was measured by the saliva-based HBGA blocking assay. As shown in Figure 6A–C, HuNoV-specific IgYs retained wildtype level of blocking ability to all three types of saliva at the temperatures below 70 °C. However, the blocking activity significantly impaired at the temperatures above 75 °C (*p* < 0.05). 

### 3.8. pH Stability of HuNoV-Specific IgY

**Figure 7:**
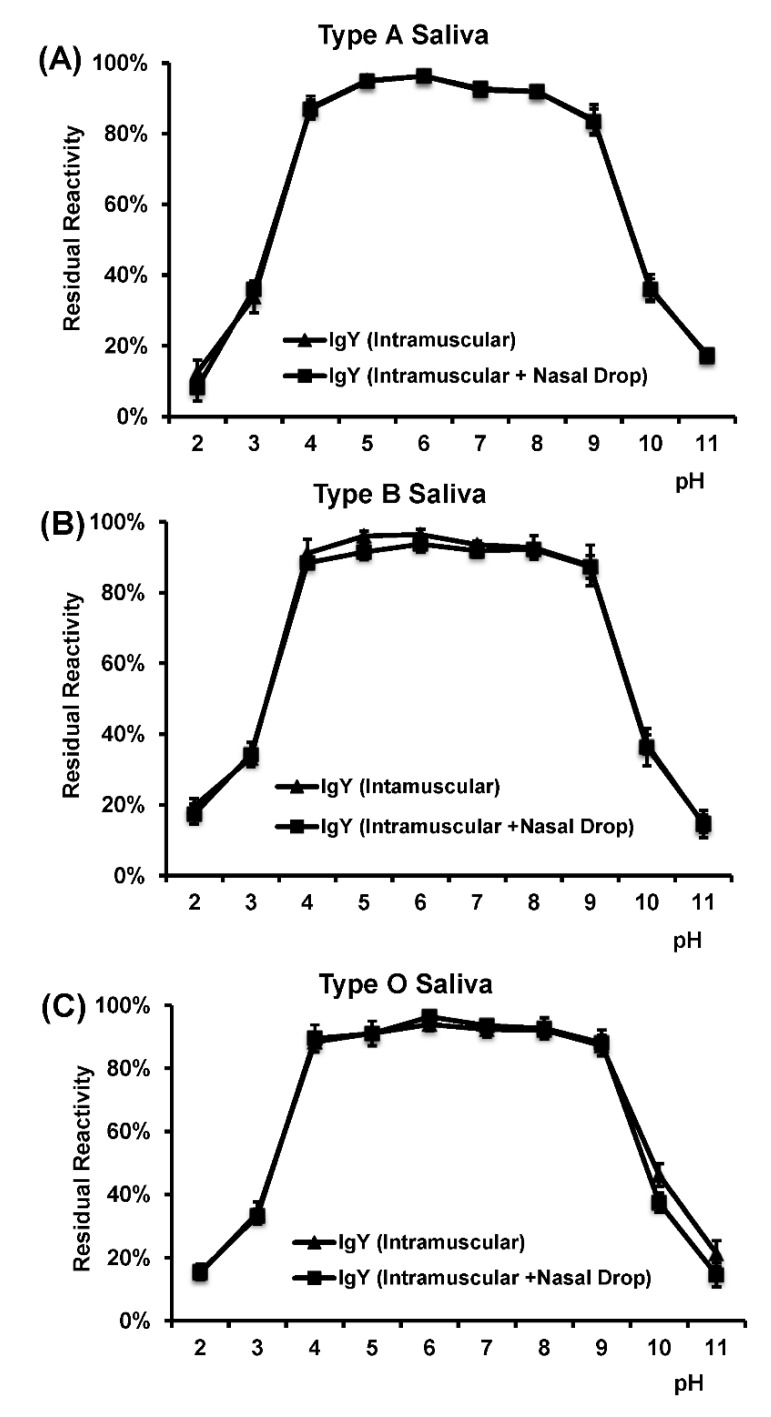
Effects of pH on the ability of IgY to block the binding of HuNoV VLPs to HBGAs. (**A**) Blockage activity to type A saliva. (**B**) Blockage activity to type B saliva. (**C**) Blockage activity to type O saliva. A percentage (%) of residual blocking activity in comparison with an untreated sample is shown. Each data point represents the average of three replicates ± the standard deviation.

Next, we determined whether HuNoV-specific IgY is stable in acid and alkaline environments. To do this, purified IgY was diluted 100 times in PBS solution and the pH of the solutions was adjusted with either HCl or NaOH to a final pH of 2–11. After incubation at 37 °C for 3 h, the solution was adjusted to neutral pH, and the receptor blocking activity was measured by HBGA blocking assay. As shown in Figure 7A–C, the blocking activity of the chicken IgY remained stable at pH 4–9 for 3 h. However, a significant decrease of blocking activity was observed when the pH is lower than 4 or higher than 9.

## 4. Discussion

In this study, we developed a highly efficient bioreactor to produce HuNoV-specific IgY in egg yolks by immunization of white leghorn chickens with recombinant rVSV-VP1. We found that intramuscular immunization alone was more efficient in triggering HuNoV-specific IgY compared to the combination of intramuscular and nasal drop immunization in hens. We demonstrated that IgY antibodies strongly reacted with HuNoV VLPs in ELISA, Western blot, and HBGA binding assays. The IgY antibodies were capable of blocking HuNoV-HBGA receptor interactions, and remained stable in temperature below 70 °C and at pH ranging from 4 to 9. Our results suggest that chicken egg yolk IgY antibodies (IgY) is a promising prophylactic and therapeutic agent for HuNoV. 

Replication competent rVSV-VP1 is an excellent antigen for production of HuNoV-specific IgY in chickens. The natural hosts of VSV are cattle, horse, deer, and pig. However, VSV has a broad tissue tropism and can replicate to a high titer in many mammalian cell lines, insect cell, yeast, worm, and *C. elegans* [30,31]. In this study, we found that rVSV-VP1 replicated to a high titer in DF-1 cells, a continuous cell line derived from chicken embryo fibroblasts. The virus yields in DF-1 cells were comparable to those produced in BSRT7 cell. In addition, rVSV-VP1 produced similar amounts of VP1 protein in both DF-1 and BSRT7 cells. Prior to our study, replication capability of VSV in avian species in vivo was poorly understood. The only report of VSV infection in chickens came from the study of rVSV-based influenza virus vaccine. It was found that chickens vaccinated with rVSV expressing the HA antigen of highly pathogenic avian influenza virus (H7N1) triggered a high level of serum neutralizing antibody and provided complete protection against lethal challenge of avian influenza H7N1 [32]. In our study, we found that hens vaccinated with rVSV-VP1 triggered a high level of HuNoV-specific IgY in yolks, further supporting that chickens were susceptible to VSV infection. We also showed that immunization route affected IgY production in hens. We found that intramuscular vaccination triggered a higher HuNoV-specific IgY than combination of intramuscular and nasal drop vaccination although the total IgY levels were similar between two vaccination routes. Our rationale to include nasal drop vaccination is that it may trigger a higher mucosal immunity since egg yolk is developed in reproductive tract of chickens. In fact, our previous mice study showed that oral and intranasal vaccination of rVSV-VP1 trigged a high level of both serum and mucosal antibody [23,24]. 

One of the major concerns of rVSV-based vaccine is the safety, particularly since VSV is neurotropic. VSV infection in ruminant animal causes vesicular lesions in the mouth, teats, and feet. In mice, wild type VSV can cause acute brain infection and fatal encephalitis [33]. However, insertion of HuNoV VP1 into VSV vector significantly attenuated the virus in a mouse model [23,24]. In this study, rVSV-VP1 did not cause any abnormal reactions, feed intake, or egg production of chickens, which further proved that rVSV-VP1 was attenuated in vivo. 

Although two recent reports showed that HuNoV-specific IgY can be induced in hen vaccinated with purified VLPs or P particles from insect cells using a baculovirus expression system [18,22], our study represents the first report of using a live attenuated recombinant virus to generate HuNoV-specific IgY. There are many potential advantages of using live attenuated rVSV-VP1 for IgY production. First, rVSV-VP1 grows to a high titer in a wide range of cell lines including chicken cells. Second, replication of rVSV-VP1 in chickens resulted in synthesis of large amount of VLPs that in turn triggered a high level of HuNoV antibody. Third, it is an economical and time-saving approach. It does not require purification of VLPs or P particles. Thus, rVSV-VP1 is a promising vaccine antigen for large-scale production of IgY in chickens. 

HuNoV-specific IgY is a potential passive immunization and therapeutic agent for HuNoV. HuNoV is a leading cause of viral gastroenteritis worldwide. Despite significant social, health, and economical burden it causes, no FDA-approved vaccine or therapeutic strategy is available. Epidemiology studies showed that HuNoV could cause lethal infection in humans, particularly in high-risk populations, such as infants, young children, the elderly, and immunocompromised individuals. Thus, there is a need to develop a safe and effective therapeutic strategy. 

We found that HuNoV-specific IgYs isolated from egg yolks were biologically functional in vitro. First, HuNoV-specific IgYs can react with VLPs in ELISA and Western blot. Second, similar to serum IgG, HuNoV-specific IgYs can be used as a primary antibody in HBGA binding assay. Third, HuNoV-specific IgYs, but not the control IgY, were capable of blocking the binding of HuNoV VLPs to type A, B, and O HBGAs in human saliva. Although it is unknown whether HuNoV-specific IgY can directly neutralize the infectious HuNoV, blockage of virus–receptor interaction will likely block the infectivity of HuNoV, which will prevent HuNoV infection and illness. In 2010, Reeck et al. found a direct correlation between the ability of an antibody to block VLP-HBGA binding and protection against HuNoV infection and illness in a HuNoV human challenge study [34]. In addition, Nurminen et al., (2011) showed that children could be protected from a GII.4 HuNoV infection due to the pre-existing HBGA blocking antibodies [35]. Thus, the IgYs is a promising passive immunization approach to prevent and treat HuNoV infection and illness. Future study will determine whether IgY can directly neutralize infectious HuNoV using the recently developed B cell culture system [8] or human enteroids culture system [9]. 

The concept of IgY passive immunization has been developed in rotavirus in vivo animal models. It was found that passive immunization of IgY could protect neonatal calves from bovine rotavirus -induced diarrhea [36]. In a mouse model, it was found that IgY could prevent murine rotavirus infection [37], bovine rotavirus-induced diarrhea [38], and human rotavirus-induced gastroenteritis [39]. Recently, human rotavirus-specific IgY administered orally as a milk supplement passively protects neonatal pigs against an enteric human rotavirus infection [40]. Porcine epidemic diarrhea virus (PEDV) is an enteric coronavirus which causes severe diarrhea, vomiting, and mortality in young piglets. Kweon et al. (2000) found that IgY passive immunization can protect piglets against PEDV infection [41]. Future experiments will investigate whether HuNoV-specific IgY can protect gnotobiotic piglets from HuNoV-induced gastroenteritis and viral shedding. 

Chicken as a “factory” for large-scale production of antigen-specific IgY. Antibody-based passive immunization and therapy has been shown to be an effective strategy to prevent infectious diseases in many animals [16]. However, preparation of serum antibody from mammals is expensive and time-consuming. Thus, large-scale application of serum antibody has been limited. IgY egg yolk immunoglobulins derived from hyperimmunized hens represent an economically feasible and practical strategy which has been explored for the passive treatment of enteric diseases. Chicken IgY production is a much easier, faster, and cheaper method for polyclonal antibody production than from any other sources. It is easy to raise chickens and collect eggs without involvement of any stressful procedures (such as bleeding). White leghorn chickens are highly productive in laying eggs, and they can continuously produce eggs containing antigen-specific antibodies in their yolks for a long time period after immunization [16]. Nguyen et al. (2010) demonstrated that chicken usually lays 280 eggs/year and each egg yolk normally contains 150–200 mg of IgY, which has 2 to 10% antigen-specific antibodies [42]. In addition, extraction of antibody from egg yolk is simple and noninvasive without affecting the immunized chickens. Therefore, a chicken is an excellent “factory” for IgY antibody production.

In order to be used as immunological supplements in infant formulas and other foods, it is important to investigate the stability of IgY during storage or following processing methods, involving thermal treatments, such as pasteurization, sterilization, or spray-drying. Based on the HBGA blocking assay, HuNoV-specific IgY remains stable at temperature below 70 °C. Thus, it is safe for pasteurization (below 72 °C) of IgY for human consumption. However, the receptor blocking activity of IgY significantly decreased when temperature reached above 75 °C, suggesting that IgY may be denatured at this temperature. For oral administration, IgY should ideally be stable in acid or alkaline environment. Our results showed that the receptor blocking activity of IgY decreased at pH below 3 or above 10. Since the stomach pH is ~2–3, it may be necessary to encapsulate the IgY in acid resistant capsules so that it can be released in intestines for neutralizing the infectious virus particles. For example, Chang et al. (1999) demonstrated that addition of sugars, glycerol, or glycine to immunoglobulin solutions was effective to protect IgYs. In addition, film coating with gum arabic was proven to be effective in reducing the degree of hydrolysis of IgY [43].

In summary, we have developed a highly efficient bioreactor to generate a high titer of HuNoV-specific IgY by vaccination of hens with rVSV-VP1. HuNoV-specific IgY was biologically active in capturing HuNoV antigen and blocking the interaction between VLPs and HBGAs. This study will facilitate the large-scale production and purification of HuNoV-specific IgY for virus detection, diagnosis, passive immunization, and therapy.
